# Associations of CAG repeat polymorphism in the androgen receptor gene with steroid hormone levels and anthropometrics among men: the role of the ethnic factor

**DOI:** 10.18699/vjgb-24-10

**Published:** 2024-02

**Authors:** L.V. Osadchuk, G.V. Vasiliev, A.V. Osadchuk

**Affiliations:** Institute of Cytology and Genetics of the Siberian Branch of the Russian Academy of Sciences, Novosibirsk, Russia; Institute of Cytology and Genetics of the Siberian Branch of the Russian Academy of Sciences, Novosibirsk, Russia; Institute of Cytology and Genetics of the Siberian Branch of the Russian Academy of Sciences, Novosibirsk, Russia

**Keywords:** AR CAG repeat polymorphism, testosterone, estradiol, anthropometrics, human male population, полиморфизм AR CAG-повторов, антропометрия, тестостерон, эстрадиол, этнические различия, популяции человека

## Abstract

Androgens are required for stimulation and maintenance of skeletal growth and bone homeostasis. Physiological functions of androgens are mediated through the androgen receptor (AR). The androgen receptor gene AR has a polymorphic trinucleotide CAG repeat and the length of AR CAG repeats determining the sensitivity of bone tissue to androgens is associated with skeleton formation and body proportions. This study aimed to investigate the relationship between AR CAG repeat polymorphism, circulating sex steroid hormones and the anthropometrics in males of different ethnic origins. Male volunteers of three ethnic groups (Slavs, Buryats, Yakuts) from urban Russian populations were recruited in a population-based study (n = 1078). Anthropometric indicators (height, arm span, leg length, the length of 2 and 4 digits of both hands) were measured and the following anthropometric indices were calculated: the ratio of height to leg length, the ratio of arm span to height, the ratio of lengths of second to fourth digit of the hand. Serum testosterone and estradiol were determined by enzyme immunoassay. Genotyping of the AR CAG repeats was performed using fragment analysis and capillary electrophoresis. Ethnic differences in all anthropometric and hormonal indicators have been established, with higher anthropometric indicators in Slavs than Buryats, and in most cases higher than in Yakuts. The testosterone level was higher among Slavs compared to Buryats, but did not differ from Yakuts; the estradiol level was lower among Slavs compared to Buryats, who did not differ from Yakuts. Buryats and Yakuts had a higher number of CAG repeats than Slavs (medians: Slavs, 23; Buryats, 24; Yakuts, 25). Positive correlations were found between the length of AR CAG repeats and estradiol levels in Buryats and testosterone levels in Yakuts, while longer CAG repeats were accompanied by higher estradiol levels in Buryats and testosterone levels in Slavs and Yakuts. Ethnic-specific correlations have been established between the steroid hormone levels and some anthropometric indicators in all ethnic groups. Available data suggest that the ethnic-specific associations of AR CAG repeats with anthropometrics can be mediated by sex steroid hormones as important regulators of skeletal growth and bone homeostasis.

## Abstract (ru)

Андрогены необходимы для роста и поддержания гомеостаза костной ткани. Физиологические функции андрогенов опосредованы андрогеновым рецептором (АR). Ген андрогенового рецептора АR имеет полиморфный тринуклеотидный CAG-повтор, и длина AR CAG-повторов, регулируя чувствительность костной ткани к андрогенам, оказывает влияние на формирование скелета и пропорций тела. Цель исследования – выявить ассоциации между длиной АR CAG-повторов, гормональными и антропометрическими показателями у мужчин различных этнических групп. В популяционном исследовании приняли участие мужчины добровольцы (n = 1078) трех этнических групп (славян, бурят и якутов) из городских популяций России. У участников измеряли рост, размах рук, длину ноги, длину второго и четвертого пальцев обеих рук, рассчитывали ростовые индексы: отношение роста к длине ноги, отношение размаха рук к росту, отношение длины второго к четвертому пальцу. Тестостерон и эстрадиол в образцах периферической крови определяли иммуноферментным методом. Генотипирование AR CAG-повторов проводили с помощью фрагментного анализа и капиллярного электрофореза. Установлены более высокие антропометрические показатели у славян по сравнению с бурятами и в большинстве случаев с якутами. Уровень тестостерона повышен у славян по сравнению с бурятами, но не отличался от якутов, а уровень эстрадиола понижен по сравнению с таковым у бурят или якутов, которые не отличались по этому показателю. Длина AR CAG-повторов составила 23, 24 и 25 триплетов (медианы) у славян, бурят и якутов соответственно. Выявлены положительные корреляции между длиной AR CAG-повторов и уровнем эстрадиола у бурят и уровнем тестостерона у якутов, причем длинные CAG-повторы сопровождались повышенным уровнем эстрадиола у бурят и тестостерона у славян и якутов. Установлены этнозависимые корреляции между уровнем стероидных гормонов и антропометрическими показателями у всех этносов. Полученные данные предполагают существование этнозависимых ассоциаций AR CAG-полиморфизма с размерами костей скелета, которые опосредуются стероидными гормонами как важными регуляторами роста и гомеостаза костной ткани.

## Introduction

Androgens, which are secreted by the Leydig cells of the testes, play a critical role in normal male physiology, and impairment of the androgen action on the target tissue is accompanied by a wide range of pathological changes. The main role of androgens consists in ontogenetic formation and maintenance of the male phenotype integrity, including a normal process of sexual differentiation, pubertal development, formation and maintenance of secondary sex characteristics, sexual behavior, and sperm production. Besides reproductive effects, androgens affect the functions of non-reproductive tissues, in particular, development and growth of the skeletal system, formation of stature and body proportions (Zitzmann, Nieschlag, 2003; Almeida et al., 2017; Alemany, 2022). Androgens exert their physiological effects on bone growth and maintenance of bone metabolism together with estrogens, which bind to the estrogen receptors present in bone tissue (Almeida et al., 2017; Alemany, 2022).

The process of bone tissue formation has several age stages. The first of them takes place in utero and is under the control of sex steroid hormones. Since the estimation of fetal androgens is complex, most investigators suggest using biomarkers, which are stable indicators reflecting the degree of exposure to androgens during fetal development. The ratio between length of the index and ring digit (2D:4D) has therefore been proposed to serve as a proxy marker for in utero androgen exposure. Several reviews and meta-analyses have shown that in most cases men and boys have lower values of this ratio than women and girls, suggesting that the gender difference in the 2D:4D ratio is determined by higher levels of androgens in male embryos compared to female (Hönekopp et al., 2007; Grimbos et al., 2010; Knickmeyer et al., 2011; Xu, Zheng, 2015; Swift-Gallant et al., 2020; Jägetoft et al., 2022). One study (Mitsui et al., 2015) showed gender differences in testosterone levels of umbilical cord blood samples, which were significantly higher in samples collected from males than those from females. These data confirm the hypothesis of sexual dimorphism with respect to the 2D:4D ratio, which reflects the levels of embryonic sex steroids. Sexual dimorphism in relation to the 2D:4D ratio persists throughout life, although data concerning sexual differences in this ratio in childhood are less variable compared to data in adults (Knickmeyer et al., 2011; Mitsui et al., 2015; Jägetoft et al., 2022).

The link found between the finger development and the prenatal androgens (and estrogens) suggests that the 2D:4D ratio is inversely related to prenatal testosterone levels and positively related to prenatal estrogen levels. There is some evidence that the 2D:4D ratio shows associations with sex steroid hormones in adults, so it can be used as a proxy marker of embryonic effects of sex steroids on a number of physiological, behavioral and anthropometric traits expressed in adults (Knickmeyer et al., 2011; Manning et al., 2014). However, the 2D:4D ratio is not always associated with the sex steroid levels in the adult male population, so the digit ratio is often the subject of debate about causality and validity as an indicator of the embryonic androgen level (Richards et al., 2020; Swift-Gallant et al., 2020).

Puberty is a unique stage of postnatal development characterized by substantial anatomical and physiological changes leading to the accumulation of bone mass, bone growth in length and the formation of a stature that is controlled by reactivation of the hypothalamic-pituitary-gonadal axis after a long period of quiet. At the beginning of puberty, androgens together with estrogens stimulate the pubertal growth spurt by increasing growth hormone (GH) secretion and hepatic insulin-like growth factor-1 (IGF-I) release, but sex steroids also have a direct effect on bone growth, since there are androgen and estrogen receptors in chondrocytes. At the end of puberty, high estrogen concentrations, but not androgens, block the longitudinal growth of bones in boys, stimulating the closure of epiphyseal growth plates, an effect mediated by the direct action of estrogens on proliferating chondrocytes (Almeida et al., 2017). Androgens are known to be precursors of estrogens in the synthetic pathways of the steroid hormones. In men, about 15 % of estradiol is secreted directly from the testes, and the remaining 85 % is derived from peripheral aromatization of androgens to estrogens by the aromatase enzyme (CYP19A1) (Almeida et al., 2017; Alemany, 2022). Estrogen resistance or aromatase deficiency in male adolescents leads to a delay in bone age and high growth, despite normal or high testosterone concentrations (Frank, 2003; Alemany, 2022).

Androgens, as well as all steroid hormones, do not affect target tissues immediately, but perform their effects essentially in the medium term, modulating gene expression; their action is relatively long and is regulated by a complex network of adaptive mechanisms in accordance with the needs of the body. Most physiological effects of androgens are mediated through the androgen receptor (AR). Since ARs are expressed in almost every tissue, “androgenicity” is manifested almost everywhere; therefore, the role of androgen receptors in males is fundamentally important (Zitzmann, Nieschlag, 2003). The androgen receptor belongs to the family of nuclear receptors of steroid and thyroid hormones and, like other members of the family, is able to interact directly with nuclear DNA. AR is a ligand-dependent nuclear transcription factor, which is activated when binding to androgens (testosterone and dihydrotestosterone) and changes the expression of AR-dependent target genes (Davey, Grossmann, 2016; Xiao et al., 2016). Interacting with certain regulatory regions, AR serves as a transcription factor regulating the synthesis of a number of proteins. There are also non-genomic effects of AR unrelated to gene expression regulation

The AR gene has a trinucleotide polymorphic CAG repeat (cytosine–adenine–guanine) in exon 1 that is transcribed into a different number of polyglutamine amino acids; that is, the variability in AR size is partially due to this trinucleotide repeat. In healthy males, the normal range of CAG repeats is 11–31 triplets, and transactivation activity of AR is inversely proportional to the number of CAG repeats (Davey, Grossmann, 2016). In vitro and in vivo studies have shown that the longer the length of CAG repeats, the weaker the transactivation ability of AR and the weaker the effects of androgens in target tissues (Buchanan et al., 2004). The authors suggest that normal function of AR is maintained in a critical and limited range of CAG repeats (16–29 triplets); the number of CAG repeats outside this range can be associated with impaired function of androgen-dependent tissues and various diseases (Davis-Dao et al., 2007; Davey, Grossmann, 2016; Ryan et al., 2017; Wang et al., 2018; Osadchuk L., Osadchuk A., 2022). It should also be noted that the testosterone effects appear after it binds to AR, affecting the transcriptional activity of AR. Thus, the AR CAG polymorphism, through reducing the sensitivity of target tissues to androgens, weakens the physiological effects of androgens and therefore is a crucial factor determining the “masculinity” of every man.

Androgen receptors are expressed in all types of bone cells (osteoblasts, osteoclasts and osteocytes). Since some polymorphic variants of AR modulate sensitivity to androgens, differences in the association between the testosterone levels and bone mass may be associated with CAG polymorphism in the AR gene. It has been established that an increased number of CAG repeats in the AR gene attenuates the testosterone effects on bone density and bone metabolism (Zitzmann, Nieschlag, 2003). In addition, a relationship was revealed between the AR CAG polymorphism and bone mineral content and bone mineral density, which was modulated by the free testosterone level (Guadalupe-Grau et al., 2010). The longitudinal growth was inversely associated with the AR CAG repeat length in boys from early prepubertal to pubertal age, but this association diminished in subsequent years and completely disappeared after the age of 16 years. The height of adult males was not associated with the AR CAG repeat length (Voorhoeve et al., 2011). The authors believe that during puberty, this relationship disappears, possibly due to the compensative increase in the activity of the hypothalamic– pituitary–gonadal axis. The effects of the AR CAG repeat length on bone mass were investigated in prepubertal boys of 12 years old (Rodríguez-García et al., 2015). In boys with longer AR CAG alleles, height, body mass, bone mineral density and content were increased compared to boys with shorter AR CAG alleles, which confirms the hypothesis that longer AR CAG alleles are associated with an increase in bone mass in prepubertal boys.

As already mentioned, we can expect a close relationship between the circulating testosterone level and the AR CAG polymorphism. Most studies have shown that the CAG repeat length directly correlates with serum testosterone levels in adult men (Crabbe et al., 2007; Huhtaniemi et al., 2009; Ma et al., 2014; Grigorova et al., 2017; Khan et al., 2018). The authors believe that longer CAG repeats impair androgen feedback in the hypothalamic-pituitary-testicular system and, thus, can increase testosterone levels. The weaker transcriptional activity of AR with a longer CAG repeat length seems to be totally or nearly totally compensated for by higher testosterone levels; therefore, an increase in testosterone levels can be considered as a compensatory effect to maintain an adequate androgen status of a man. From a genetic perspective, the testosterone level is undoubtedly a polygenic trait, and the AR CAG polymorphism is just one of many genetic factors underlying the genetic control of this trait. Individual variation in testosterone levels in healthy men can be explained by the AR CAG polymorphism. In a large study population of healthy Belgian men, it was shown that the CAG repeat length was positively associated with serum total testosterone (Crabbe et al., 2007). The authors suggest that in men, 6.0–8.5 % of individual testosterone variability can be associated with the CAG repeat length in the AR gene

In our previous work, it was shown that the variability of the AR CAG repeat length was associated with the ethnic composition of the studied population (Osadchuk et al., 2022). Significant differences were observed in the AR CAG repeat length between the most common ethnic cohorts of Slavs (Caucasians), Buryats (Asians), and Yakuts (Asians) with median in Slavs – 23; Buryats – 24; Yakuts – 25. The Slavs have the largest range (7–36 repeats), the Yakuts have the smallest range (18–32 repeats) and the Buryats have the middle range (11–39 repeats). Longer CAG repeats were associated with an impaired semen quality within the Slav and Buryat groups, but this effect was not found in Yakuts

Based on the above, we can expect there to be an effect of the AR CAG polymorphism on the variability of the sex steroid hormone levels in Russian men, which may affect the formation of some androgen-dependent anthropometric parameters. The aim of this study was to study the possible relationship between variations in the AR CAG repeat length, which affects the transactivation activity of AR, and circulating sex steroid hormones, as well as with steroid-dependent anthropometric parameters. In addition, it was interesting to compare the above associations in men of different ethnicity, who also differ in the AR CAG repeat length, anthropometric indicators, testosterone and estradiol levels. To achieve these goals, a multicenter population study of Russian male volunteers was conducted, in which the AR CAG repeat length, serum testosterone and estradiol levels, anthropometric indicators, including height, leg length, arm span, the 2nd to 4th digit ratio of both hands were determined. To clarify the role of the ethnic factor, the study was conducted on men of three ethnic groups – Slavs, Buryats and Yakuts, who had previously been studied with respect to the AR CAG polymorphism (Osadchuk et al., 2022). It should be noted that this is the first Russian study clarifying the role of the AR CAG polymorphism in regulating sex steroid hormone levels and steroid-dependent anthropometric indicators.

## Materials and methods

Young male volunteers (n = 1078) of three ethnicities from five Russian cities participated in this study: Slavs from Archangelsk, Novosibirsk, Kemerovo; Buryats from Ulan- Ude, Yakuts from Yakutsk. The Slavic group consisted of Russians (95.9 %), Ukrainians (0.5 %) and descendants of mixed marriages of Russians, Belarusians, Ukrainians (3.5 %). The men filled out questionnaires, including questions about age, place of birth, nationality, profession, work conditions, noted military service, smoking and alcohol consumption, past and current diseases. Ethnicity of participants was obtained according to the information from the self-reporting questionnaires, taking into account ethnicities of the participants’ parents and grandparents. As a rule, the participants considered themselves healthy and had not previously consulted doctors about chronic diseases. The inclusion criteria for participation in the study were the absence of acute diseases or taking medications that affect hormone levels or bone metabolism. All participants gave informed consent for participation. The ethics committee of the Federal Research Center “Institute of Cytology and Genetics”, the Siberian Branch of the Russian Academy of Sciences, approved the study (Protocol No. 160, date 17.09.2020).

Height, body weight, waist and hip circumference (WC and HC, respectively), leg length, arm span, length of index and ring fingers of both hands were measured in all participants. Body mass index (BMI), trochanteric index (TI), androgenic deficiency index (ADI) and digital index (2D:4D right and left) were calculated. Body weight was estimated in kg, WC, HC, height, leg length, arm span in cm. BMI is the main anthropometric indicator of obesity and is calculated as the ratio of body weight (kg) / height (m2). TI characterizes the body proportion formed by the end of puberty and is calculated as the ratio of height to leg length. ADI also characterizes the proportions of the body formed by the end of puberty and is calculated as the ratio of arm span to height. This indicator depends on the testosterone level in adolescents, and if androgen deficiency took place before puberty, then the arm span begins to exceed the height and a specific tallness with eunuchoid body proportions is formed (Frank, 2003). The length of 2D and 4D was measured by digital caliper; the 2D:4D ratio was calculated as the ratio of the length of 2 finger to 4 finger. It is assumed that the 2D:4D ratio reflects the prenatal androgenization and does not change during postnatal life (Knickmeyer et al., 2011; Manning et al., 2011).

Fasting blood samples from the cubital vein were drawn in the morning between 8–11 hr. Blood samples were centrifuged in 15–20 min and at 1500 rpm; serum was stored at −40 °С until hormonal analysis. Serum concentrations of luteinizing hormone (LH), follicle-stimulating hormone (FSH), testosterone (T); estradiol (E2) were determined by enzyme immunoassay with commercially available kits (Alkor Bio, Xema, Russia) according to the manufacturer manuals. The ranges of evaluated concentrations for our study population were as follows: LH – 1.3–6.7 mIU/ml; FSH – 1.3–8.8 mIU/ml; Т – 11.7–38.2 nmol/L; Е2 – 0.10–0.35 nmol/L.

This study included men who had previously been genotyped for AR CAG repeats; the genotyping technique was described in detail earlier (Osadchuk et al., 2022). Briefly, genomic DNA was extracted from peripheral blood leukocytes using the common phenol-chloroform method. Genotyping of the AR CAG repeats was performed by the method of fragment PCR analysis and capillary electrophoresis using the sequencer “Nanophor-05” (Syntol, Russia). The method allows to determine the relative length of the product in relation to the length standard and is based on the separation of DNA into fractions by molecular weight. The primer sequences were as follows: forward, 5′-(FAM)-TCCAGAATCTGTTCC AGAGCGTGC-3′ and reverse, 5′-GCTGTGAAGGTTGCT GTTCCTCAT-3′. The number of CAG repeats was calculated using an allelic ladder of marker fragments, which consisted of eight fragments of different lengths and were used as an internal standard for calculations (lengths of CAG repeats were 12, 19, 21, 23, 25, 27, 29, 33 triplets).

The statistical analysis of the data was performed using the statistical package “STATISTICA” (version 8.0). The results are presented as mean (SD). The Kolmogorov-Smirnov test for normality was used. Since most parameters were not normally distributed, the Kruskal–Wallis ANOVA test for comparing multiple independent groups was carried out to find the differences in the hormonal and anthropometric parameters among ethnic groups. Spearman correlation coefficients were used to determine correlations among parameters in each ethnic group. Duncan’s test was used for pairwise comparison of groups. The testosterone and estradiol levels were best normalized by log transformation before analysis.

The next step to find out possible associations between the AR CAG polymorphism and hormonal and anthropometric indicators was the stratification of participants in each ethnic group into three CAG categories based on the CAG ethnic range restriction with a frequency below 5 % for short or long CAG repeat length. The rest of the CAG repeat range represented the medium CAG repeat category. The categorization of the CAG repeat length corresponded to the one presented earlier (Osadchuk et al., 2022). The results of the stratification of participants by the CAG repeat length (number of triplets) are as follows: Buryats – short (≤ 20); medium (21–27); long (≥ 28); Slavs – short (≤ 19); medium (20–24); long (≥ 25); Yakuts – short (≤ 22); medium (23–27); long (≥ 28). Differences in anthropometric and hormonal variables between subgroups with different CAG repeat length were tested by analysis of covariance (ANCOVA); hormonal variables were adjusted for age, body weight, WC, BMI. A p-value ≤ 0.05 was regarded as statistically significant.

## Results

Ethnic differences in anthropometric and hormonal parameters, and the AR CAG repeat length. The ethnic groups differed in age; the Buryats were 1.5 years younger than the Slavs and the Yakuts ( р ≤ 0.05), who did not differ from each other (Table 1). Almost all anthropometric indicators were noted to be higher in Slavs compared to Buryats ( р ≤ 0.05), with the exception of lower values of TI and the length of the ring finger in Slavs ( р ≤ 0.05). The Slavs differed from the Yakuts by higher values of almost all anthropometric indicators ( р ≤ 0.05), but did not differ in WC and length of both index fingers, and were characterized by lower TI, ADI and the length of both ring fingers ( р ≤ 0.05, see Table 1). The Buryats did not differ from the Yakuts in most anthropometric indicators, with the exception of higher height, TI and lower WC and ADI ( р ≤ 0.05). Thus, the Buryats and the Yakuts are closer to each other in body proportions and are significantly differentiated from the Slavs

**Table 1.**
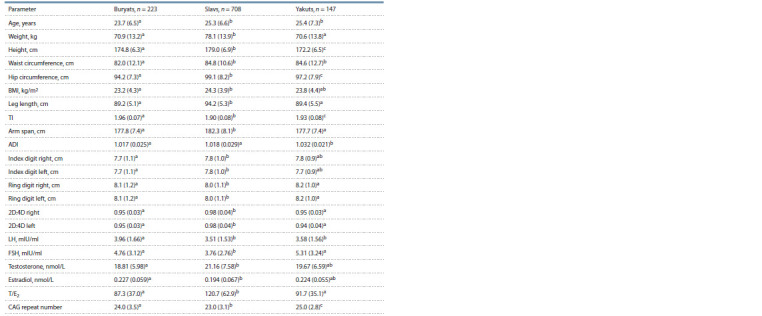
Anthropometric and hormonal parameters, and AR CAG repeat length in men of three ethnic groups Notе here and further. Values are presented as mean (SD); BMI, body mass index; TI, trochanter index (the ratio of height to leg length); ADI, androgen deficiency index (the ratio of the length of arm span to height); 2D:4D-right, 2D:4D-left (the ratio of the length of second finger to fourth); LH, luteinizing hormone; FSH, follicle-stimulating hormone; Т/Е2, the ratio of testosterone to estradiol concentrations; a, b, c comparisons with different superscripts within variables were significant ( р ≤ 0.05).

The serum levels of LH, FSH and estradiol were lower, and the testosterone level and the T/E2 ratio were significantly higher in Slavs compared to Buryats ( р ≤ 0.05, see Table 1). Buryats and Yakuts did not differ from each other in all hormonal parameters, with the exception of lower LH values in Yakuts ( р ≤ 0.05). Thus, the Buryats and the Yakuts were close to each other in hormonal status and more differentiated from the Slavs. The length of CAG repeats significantly differed between all ethnic groups (medians: Slavs – 23; Buryats – 24; Yakuts – 25, р ≤ 0.05).

Correlations between the AR CAG repeat length, hormonal and anthropometric indicators in men of three ethnic groups. The relationships between the CAG repeat length, hormonal and anthropometric indicators were determined by ethnic origin (Table 2). In Buryats, a positive correlation was detected between the CAG repeat length and the 2D:4D ratio right, as well as the estradiol level ( р ≤ 0.05), in Yakuts – between the CAG repeat length and the testosterone level ( р ≤ 0.05), and in Slavs, no reliable correlations were found (see Table 2). More numerous correlations were observed between hormone levels and anthropometric characteristics, which were also modulated by ethnic origin (see Table 2). In Buryats, negative correlations were established between estradiol level and arm span, and ADI ( р ≤ 0.05); in Yakuts – positive correlations between estradiol level and leg length, and lengths of all four digits ( р ≤ 0.05), but negative correlation between the estradiol level and TI ( р ≤ 0.05). The Slavs had a positive correlation between testosterone level and leg length, as well as both 2D:4D ratios ( р ≤ 0.05); a negative correlation between testosterone level and TI, and lengths of all four digits ( р ≤ 0.05). In addition, positive correlations were noted between estradiol level and lengths of all four digits (see Table 2, р ≤ 0.05).

**Table 2.**
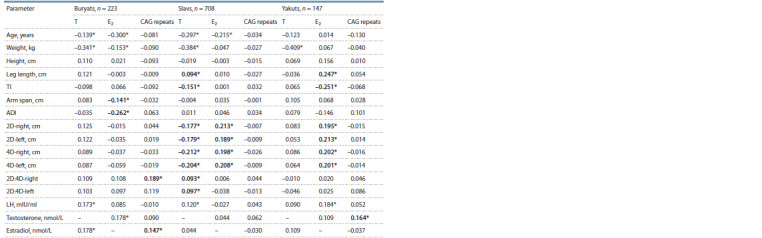
Correlations between anthropometric and hormonal parameters, and AR CAG repeat length in men of three ethnic groups (Spearman’s test) Correlation is significant ( p <0.05).

Anthropometric and hormonal parameters in subgroups with different AR CAG repeat lengths in men of three ethnic groups. Anthropometric and hormonal data in the subgroups of short, medium and long CAG repeats in each ethnic group are presented in Table 3. The Buryats had significant differences in height, ADI, the 2D:4D ratio right, estradiol level between the subgroups with short and long CAG repeats ( р ≤ 0.05), the Slavs – in leg length and testosterone level ( р ≤ 0.05), the Yakuts – in arm span, ADI, testosterone level ( р ≤ 0.05). Thus, in all ethnic groups, long CAG repeats were accompanied by an increased level of steroid hormones.

**Table 3.**
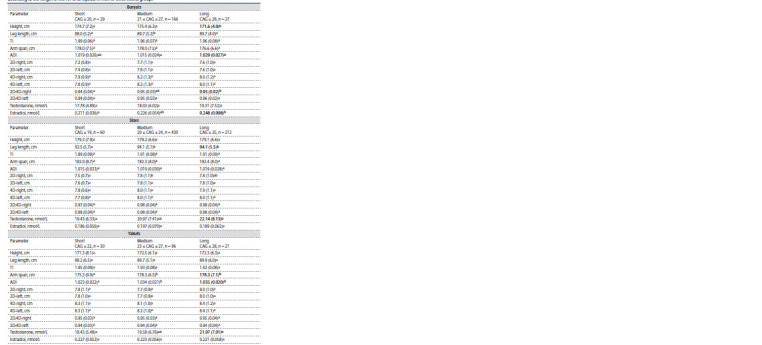
Correlations between anthropometric and hormonal parameters, and AR CAG repeat length in men of three ethnic groups (Spearman’s test) Correlation is significant ( p < 0.05).

## Discussion

Comparison of anthropometric and hormonal indicators in young Russian men of different ethnicity allowed us to establish reliable ethnic differences in various anthropometric indicators and the levels of sex steroid hormones. Attention is drawn to the higher anthropometric indicators that determine the male stature (height, leg length, arm span) in the Slavs than the Buryats, and in most cases, than the Yakuts, which corresponds to a higher current testosterone level and a lower current estradiol level in the Slavs compared to the Buryats (the Yakuts occupied an intermediate position). The established hormonal differences indicate ethnic features in the functioning of the hypothalamic-pituitary-testicular axis and suggest that they may have formed during the embryonic or pubertal periods, and led to ethnic differences in hormonedependent anthropometric characteristics in adults. We found a negative correlation between estradiol levels and arm span in Buryats, a positive correlation between estradiol levels and leg length in Yakuts, and a positive correlation between testosterone levels and leg length in Slavs. Thus, it seems that in Buryats and Yakuts, estradiol acts as a determinant of the longitudinal growth of the skeleton, determining the stature, while in Slavs testosterone performs this function. Based on these facts, it can be assumed that in adolescence in Buryats and Yakuts, increased estradiol levels and reduced testosterone levels, unlike in Slavs, can contribute to earlier closure of the epiphyseal plates of tubular bones, thereby stopping their further growth and delaying the growth of the skeleton.

The length of AR CAG repeats was the shortest among the Slavs, higher among the Buryats and the highest among the Yakuts. Positive correlations between the AR CAG repeat length and the estradiol levels in Buryats or the testosterone levels in Yakuts were supplemented by the association of long CAG repeats with increased estradiol levels in Buryats and increased testosterone levels in Yakuts and Slavs. Thus, it were the long CAG repeats that coordinated the variability of steroid hormone levels in all three ethnic groups. Taking into account the role of sex steroid hormones as important regulators of skeletal bone growth and bone tissue homeostasis, it can be assumed that sex steroid hormones can mediate the ethno-specific effects of AR CAG repeats on male anthropometric characteristics. Moreover, the AR CAG repeat polymorphism can predict the sex steroid level in men of the ethnic group studied

In the current paper, the identified effects of long AR CAG repeats on the steroid hormone levels coincide with those previously obtained in European men and generally confirm that individual variability of testosterone and/or estradiol levels in men may be partially due to the AR CAG repeats polymorphism (Crabbe et al., 2007; Huhtaniemi et al., 2009; De Naeyer et al., 2014). In aging men from 8 European countries, AR CAG repeat length positively correlated with serum testosterone and estradiol levels, while higher testosterone levels in men with long AR CAG repeats corresponded to a lack of age-related hypogonadism in these patients (Huhtaniemi et al., 2009).

Hormonal effects of the AR CAG polymorphism have not been confirmed in Filipino men (Ryan et al., 2017) and Greek men (Goutou et al., 2009). The discrepancy in the results may be a consequence of ethno-specific characteristics or mixed ethnic composition of the studied groups. It should be noted that lifestyle factors (obesity, physical inactivity, taking anabolic steroids, stress, etc.) can affect the hormonal background, and altered levels of testosterone or estradiol will mask the genetic effects of the AR CAG polymorphism in men (Wrzosek et al., 2020).

Studies in vitro and in vivo demonstrated that the longer the length of AR CAG repeats, the weaker the transactivation ability of AR and the weaker the effects of androgens in target tissues. A possible mechanism underlying this phenomenon may be related to specific proteins known as coregulators that modulate the transcriptional activity of androgen-bound AR (Buchanan et al., 2004; Davey, Grossmann, 2016). In Slavs and Yakuts, an increase in the CAG repeat length is accompanied by an increase in testosterone levels, which compensates for a decrease in AR functional activity. In addition, in the hypothalamic-pituitary-testicular system, longer AR CAG repeats weaken androgen feedback and increase testosterone levels (Huhtaniemi et al., 2009). From a genetic point of view, the AR CAG polymorphism is one of many genetic factors underlying the genetic control of testosterone levels as a polygenic trait.

The normal AR function is maintained in a critical and limited range of CAG repeat lengths. Molecular modeling revealed a critical range of 16–29 triplets that would maintain maximum interaction between the transactivating domain and hormone binding domain of the AR (Nenonen et al., 2010). Consequently, the analysis of CAG repeat length in linear regression models, performed in most studies, is probably not adequate enough, and data stratification may be an alternative way to study the relationship of CAG repeat length with phenotypic traits. Indeed, in our study, we failed to establish the linear relationship between the AR CAG repeat length and the steroid hormone level in the Slavic group, however, when stratifying CAG repeats into short, medium and long, the effects of long CAG repeats were revealed. A similar way has been successfully applied in other studies that have established the stimulating effects of long AR CAG repeats on a number of hormonal and anthropometric traits, including the level of total and/or free testosterone and/or estradiol (Crabbe et al., 2007; De Naeyer et al., 2014; Khan et al., 2018), as well as height, body weight, bone mineral density in adolescent boys (Rodríguez-Garcí et al., 2015).

As already mentioned, the testosterone effect on bone growth and metabolism is exerted together with estradiol after the conversion of testosterone into estradiol by the aromatase enzyme, and is mediated by estrogen receptors ERα, ERβ (Almeida et al., 2017; Alemany, 2022). Probably, in Buryat carriers of long AR CAG repeats, the estrogen effect on some anthropometric indicators (height, ADI, the 2D:4D ratio) may be due to the increased aromatase activity involved in the action of androgen and AR on bone growth.

The variability of the AR CAG repeat length associated with testosterone levels can already affect anthropometric parameters in embryogenesis, including finger length and the 2D:4D ratio (Manning et al., 2002; McIntyre, 2006; Grimbos et al., 2010; Knickmeyer et al., 2011; Folland et al., 2012). In our study, the CAG repeat length positively correlated with the 2D:4D ratio, and long AR CAG repeats increased the 2D:4D ratio in Buryats; it could probably be due to lower prenatal androgenization. However, we found a positive relationship between testosterone levels and both 2D:4D ratios in Slavic men, which does not seem to be associated with long AR CAG repeats. It is worth noting that the validity of the 2D:4D ratio remains controversial, since the research data are very contradictory. There are studies both confirming and not confirming the relationship of the digit index with the level of testosterone and/or estradiol or with the CAG repeat length in adult men (Hönekopp et al., 2007; Knickmeyer et al., 2011; Muller et al., 2011; Hönekopp, 2013; Zhang et al., 2013). In animal studies, there is more reliable experimental evidence of the effect of prenatal sex hormones on the 2D:4D ratio and its relation to the AR CAG repeat length (Zheng, Cohn, 2011; Swift-Gallant et al., 2020). Our results complement the existing studies, but indicate the ethnic characteristics of such associations.

## Conclusion

The study found 1) differences in hormonal and anthropometric indicators between men of three ethnic groups: Buryats, Yakuts and Slavs; 2) ethno-specific correlations between hormonal and anthropometric indicators; 3) ethno-specific positive correlations between the AR CAG repeat length and sex steroid levels; 4) ethno-specific effects of long AR CAG repeats on selected anthropometric traits and sex steroid levels in all ethnic groups.

## Conflict of interest

The authors declare no conflict of interest.
